# Effects of a second iron injection and sire line on growth performance, hemoglobin levels, antioxidative status, and whole-body iron retention in piglets

**DOI:** 10.1093/jas/skag192

**Published:** 2026-06-23

**Authors:** Jannell Torres, Chanho Kwon, Eva Safaie, Savannah Locke, Madison Mejia, Shelby Greer, Jihye Lee, Yoon Soo Song, Wesley Lyons, Chris Olsen, Scott Fritz, Young Dal Jang

**Affiliations:** Department of Animal and Dairy Science, University of Georgia, Athens, GA 30602, United States; Department of Animal and Dairy Science, University of Georgia, Athens, GA 30602, United States; Department of Animal and Dairy Science, University of Georgia, Athens, GA 30602, United States; Department of Animal and Dairy Science, University of Georgia, Athens, GA 30602, United States; Department of Animal and Dairy Science, University of Georgia, Athens, GA 30602, United States; Department of Animal and Dairy Science, University of Georgia, Athens, GA 30602, United States; Department of Animal and Dairy Science, University of Georgia, Athens, GA 30602, United States; Department of Animal and Dairy Science, University of Georgia, Athens, GA 30602, United States; Pharmacosmos Inc, Watchung, NJ 07069, United States; Pharmacosmos Inc, Watchung, NJ 07069, United States; Kansas Veterinary Diagnostic Laboratory, Kansas State University, Manhattan, KS 66506, United States; Department of Animal and Dairy Science, University of Georgia, Athens, GA 30602, United States

**Keywords:** antioxidant capacity, hemoglobin, iron injection frequency, iron store, piglets, sire line

## Abstract

A total of 156 newborn piglets from 15 sow litters (initial body weight [BW] of 1.75 ± 0.38 kg at d 3 of age; seven from PIC337 and eight from PIC800 sire lines, all bred to Camborough sows) were allotted to six treatments in a 2 × 3 factorial arrangement. Main factors were: 1) sire line (PIC337 vs. PIC800), and 2) number of iron injections (no, one, or two intramuscular injections of 200 mg iron) administered at d 3 (one injection), or d 3 and 8 (two injections) of age. Six pigs per sire line at birth and six per treatment at weaning (d 18.9 ± 1.6 of age) were euthanized for various organs, whole blood and remaining body tissue collection for iron analysis. Remaining weaned pigs were transferred to a nursery facility and fed a common diet for 28 d. Growth performance, hemoglobin levels, antioxidant status, and iron retention were measured. Although there was no difference in pre-weaning performance between two sire lines, PIC337 pigs tended to have greater overall nursery feed intake (*P *= 0.10) and BW at d 28 postweaning (*P *= 0.06) than PIC800 pigs. PIC337 pigs had lower hemoglobin levels than PIC800 pigs from d 15 of age to d 7 postweaning (*P *< 0.11), while no differences were observed in blood antioxidant parameters. Increasing iron injection frequency linearly increased BW at weaning and d 28 postweaning and overall nursery weight gain (*P *< 0.05), while increasing hemoglobin levels linearly and quadratically from d 15 of age to d 14 postweaning (*P *< 0.05). Plasma malondialdehyde levels increased quadratically at weaning (*P *= 0.06) and d 14 postweaning (*P *< 0.05) with increasing iron injection frequency, while plasma catalase level increased linearly at d 14 postweaning (*P *< 0.05). Liver iron mass at birth was greater in PIC800 pigs than in PIC337 pigs (*P *< 0.05), while no differences were observed between sire lines in whole-body iron mass at birth and weaning, or in iron content of colostrum and milk. Total iron mass in individual organs and whole-body of pigs at weaning (*P *< 0.05) increased linearly with increasing iron injection frequency. In conclusion, increasing iron injection frequency before weaning improved pre- and post-weaning growth, hemoglobin levels, plasma catalase levels, and whole-body iron retention, confirming that most of the injected iron was retained in the body, while hemoglobin levels were influenced by the sire lines without affecting overall body iron retention.

## Teaser

Increasing iron injection frequency before weaning improved piglet growth, hemoglobin levels, antioxidant capacity, and iron stores, while no iron injection caused iron deficiency, reduced growth and antioxidant capacity, with hemoglobin levels varying by genetic lines but overall iron retention remaining similar between sire lines.

## Introduction

Newborn pigs are susceptible to iron deficiency anemia due to low body iron reserves at birth, which are only sufficient to meet requirements for up to d 3–4 after birth (Szudzik et al. 2018), and low iron content in sow milk (Lipinski et al. 2010). Thus, a single intramuscular (IM) injection of 200 mg iron within the first 5 d after birth is a common practice in swine production (Murphy et al. 1997; Szudzik et al. 2018). However, It has been reported that a second 200 mg iron injection during the suckling period improved hemoglobin levels and post-weaning growth performance compared with a single 200 mg injection (Chevalier et al. 2023; Johnson et al. 2025). This indicates that a single injection may be insufficient to maintain appropriate hemoglobin levels through weaning, and pigs may require additional iron administration to meet their iron needs in the suckling and nursery periods (Heidbüchel et al. 2019; Johnson et al. 2025).

In pigs, as iron is deposited mainly in the liver, hepatic iron content can be indicative of whole-body iron (Ganz and Nemeth 2012). As pigs do not possess a regulated pathway for excretion, once iron is administered, it remains in the body unless major blood loss occurs (Correnti et al. 2024). Previous studies reported that pigs receiving an additional iron injection resulted in greater iron content in major organs such as the liver, spleen, heart, and kidney (Chevalier et al. 2021; Johnson et al. 2025). However, there is limited information about iron retention in all important organs and whole-body tissue in pigs administered different levels of parenteral iron.

Furthermore, modern advances in swine genetics have produced larger litters and faster-growing pigs, potentially increasing iron requirements postnatally as body iron status depends heavily on growth-related increases in blood volume and tissue accretion (Szudzik et al. 2018).  Egeli and Framstad (1998) reported that Duroc and Landrace pigs receiving a 180 mg iron injection showed inter-breed differences in hemoglobin levels at weaning. In addition, Chevalier et al. (2024) reported that herd health status, genetic background, and management practices can influence pigs’ hematological responses to additional iron administration. Thus, understanding whole-body iron retention and hematological response to iron injection for suckling pigs are crucial for accurate determination of their iron requirements, particularly when evaluating iron injection frequency or dosage in modern genetic lines.

Therefore, the objective of the current study was to evaluate the effects of IM iron injection frequency during the suckling period on growth performance, hematological parameters, antioxidant status, and whole-body iron retention in pigs from two different sire lines. 

## Materials and methods

### Animal use protocol

This experiment and sample collections were carried out in an environmentally controlled nursery facility at the University of Georgia swine farm (Winterville, GA) and University of Georgia Large Animal Research Unit (Athens, GA). The experiment was conducted under protocol (#A2024 06-007-Y1-A0) approved by the Institutional Animal Care and Use Committee of the University of Georgia.

### Animals, experimental design, and housing

A total of 15 Camborough sows were bred using semen from either PIC337 (seven sows; average parity 4.14 with average number of liveborn of 11.43) or PIC800 (eight sows; average parity 3.38 with average number of liveborn of 12.75). During gestation, the sows were housed in a group housing system equipped with electronic sow feeders and were fed 2–2.5 kg of a standard gestation diet (3,330 kcal metabolizable energy/kg, 17.4% crude protein, 0.7% total lysine, and 110 ppm of supplemental iron) based on body weight (BW) and parity following the University of Georgia swine farm protocol. On d 107–110 of gestation, all sows were moved to farrowing crates and provided a common lactation diet containing 3,300 kcal metabolizable energy/kg, 22.4% crude protein, 1.10% total lysine, and 110 ppm of supplemental iron offered ad libitum throughout the lactation period.

At farrowing, six piglets per sire line (three boars and three gilts; total 12 pigs) that had average BW within each sire line and litter (one boar and one gilt from each of three litters) were selected and euthanized before colostrum intake for tissue collection (average BW: 1.45 ± 0.20 kg and 1.42 ± 0.18 kg for PIC337 and PIC800 groups, respectively). At d 3 of age, a total of 156 pigs (initial BW 1.75 ± 0.38 kg d 3 of age; Camborough × PIC337 and Camborough × PIC800) from 15 sows were selected and allotted to three treatments within the litter in a 2 × 3 factorial arrangement (main factors were the sire line and number of iron injections) based on BW and sex and housed in the farrowing crate along with the sow, with a heat lamp provided. All pigs were processed based on the standard protocol of the University of Georgia swine farm. Pig sets with three pigs in each set were created by having six to nine littermates within the same sex that had similar BW within sire line (PIC337 or PIC800). Within each set, one pig was assigned to: no, one, or two iron injection treatment. Treatments were: 1) Control (C)-PIC337: pigs born from sire line PIC337 + no iron injection, 2) IRON-PIC337: IM iron injection (200 mg iron) at d 3 of age, 3) 2IRON-PIC337: two IM iron injections (200 mg iron for each) at d 3 and 8 of age, 4) C-PIC800: pigs born from sire line PIC800 + no iron injection, 5) IRON-PIC800: IM iron injection (200 mg iron) at 3 d of age, and 6) 2IRON-PIC800: two IM iron injections (200 mg iron for each) at d 3 and 8 of age. A common iron-dextran product (UNIFERON 200, Pharmacosmos, Inc., Watchung, NJ) was used for both injections. The first iron injection was administered into the right neck muscle, and the second iron injection was administered into the left neck muscle. No creep feed was provided for the piglets in the entire suckling period. There were 11 and 14 piglets that died from PIC337 and PIC800 groups in the suckling period, respectively, due to reasons unrelated to treatment which were excluded from the data set.

At weaning, six pigs per treatment (three barrows and three gilts; total 36 pigs) that had average BW within treatment and litter (three barrows from each of three litters and three gilts of three litters selected within each sire line) were selected and euthanized for tissue sample collection (average BW: 5.03 ± 0.58 kg and 5.07 ± 0.0.35 kg for PIC337 and PIC800 groups, respectively). Then, remaining piglets after tissue collection were moved to the nursery facility at the University of Georgia Large Animal Research Unit. Due to unequal number of weaned pigs between PIC337 and PIC800 groups, 66 pigs (11 pigs per iron injection treatment per each sire line) were selected within set to match average weaning weight out of 95 remaining pigs and allotted to nursery pens (1 m × 2 m) in three replicates balanced by BW and sex with four pigs per pen in the first two replicates and three pigs per pen in the last replicate for a 28-d post-weaning growth period. Pigs were raised with free access to water and feed in the environmentally controlled nursery facility. All pigs were fed common corn-soybean meal-based nursery diets with dicalcium phosphate and limestone that met or exceeded nutrient requirement of NRC (2012) with supplemental 110 mg of iron/kg diet. Two diet phases were used for d 0–14 (Phase 1) and d 14–28 (Phase 2) postweaning. Basal diet samples were analyzed for iron content at Agricultural Experimental Station Chemical Laboratories in the University of Missouri-Columbia (Columbia, MO). Diet formulation, calculated chemical composition, and analyzed iron content were presented in Table 1.

**Table 1:** Diet formulation and calculated chemical composition.^1^

Ingredient, %	Phase 1 (d 0–14 postweaning)	Phase 2 (d 14–28 postweaning)
** Corn**	53.92	60.25
** Soybean meal (dehulled)**	22.00	26.00
** Whey (dried)**	10.00	5.00
** HP300^2^**	5.00	3.50
** Fish meal**	3.50	–
** Soybean oil**	2.00	1.50
** l-Lysine**	0.45	0.43
** dl-Methionine**	0.24	0.21
** l-Threonine**	0.24	0.21
** l-Tryptophan**	0.05	0.03
** l-Valine**	0.10	0.07
** Dicalcium phosphate**	0.75	0.95
** Limestone**	0.85	0.95
** Salt**	0.50	0.50
** Vitamin mix^3^**	0.25	0.25
** Trace mineral mix^4^**	0.15	0.15
**Calculated chemical composition**		
** Metabolizable energy, kcal/kg**	3,241	3,370
** Crude protein, %**	22.03	20.71
** SID Lys, %**	1.40	1.28
** SID Met+Cys, %**	0.83	0.76
** Toal Ca, %**	0.80	0.70
** STTD P, %**	0.40	0.33

SID, standardized ileal digestible; STTD, standardized total tract digestible.

1 Analyzed iron content in Phases 1 and 2 were 198 and 200 ppm, respectively.

2 Hamlet Protein, Findlay, OH.

3 Vitamin premix supplied the following per kilogram of diet: 11,000 IU of vitamin A, 1,600 IU of vitamin D_3_, 99 IU of vitamin E, 4.4 mg of vitamin K, 55 µg of vitamin B_12_, 9.9 mg of riboflavin, 31.9 mg of pantothenic acid, 55 mg of niacin, 0.9 mg of folic acid, 3.9 mg of vitamin B_6_, 3.1 mg of thiamin, and 0.3 mg of biotin, 600 mg of choline chloride.

4 Trace mineral premix supplied the following per kilogram of diet: 33 mg of Mn as manganous oxide, 110 mg of Fe as ferrous sulfate, 110 mg of Zn as zinc sulfate, 16.5 mg of Cu as copper sulfate, 0.3 mg of I as Ca iodate, 0.3 mg of Se as sodium selenite.

### Data and sample collection and processing

In the suckling period, the BW of each pig was recorded at d 0, 3, 8, and 15 of age, and weaning (d 18.9 ± 1.6 of age) and average daily gain (ADG) were calculated during the suckling period. To account for differences in gestation length between sire lines resulting in unequal weaning age between sire lines, weaning weight and weight gain were adjusted to a uniform weaning age, following the method of Jang et al. (2017). A drop of blood from the ear veins of each pig was loaded into disposable microcuvettes via capillary action and analyzed for hemoglobin levels at d 3, 8, and 15 of age, and weaning using the HemoCue Hb 201^+^ System (HemoCue, Brea, CA). Colostrum and milk samples were collected from each sow within 24 h postpartum and at d 15 of lactation, respectively, after injecting 10 IU of oxytocin via ear vein, and stored at −20 °C for further iron analysis.

In the nursery period, BW of each piglet and pen feed consumption were recorded at d 0, 7, 14, 21, and 28 postweaning for calculation of ADG, average daily feed intake (ADFI), and gain-to-feed ratio (G:F). Hemoglobin levels were measured using the HemoCue Hb 201^+^ System at d 0, 7, 14, 21, and 28 postweaning, as described above. Blood samples were collected from piglets (one barrow and one gilt per pen having pen average BW) from jugular vein into K_3_EDTA tubes at weaning, d 14, and 28 postweaning. Plasma samples were separated by centrifugation at 2,500 × *g* for 30 min at 4 °C and stored at −80 °C until analysis.

At farrowing, six piglets per sire line (total 12 pigs) before colostrum intake and at weaning, six pigs per treatment (total 36 pigs) that had average BW within treatment were selected and euthanized using a CO_2_ method by trained personnel, followed by exsanguination for tissue sample collection of the heart, kidney, liver, and spleen. For collection of whole blood during exsanguination at farrowing, piglets were suspended over a collection vessel. To prevent blood clotting, 6 mL of 3.8% sodium citrate was added to the collection vessel. At weaning, 20 mL of 3.8% sodium citrate was added to the collection vessel to prevent blood clotting. Collection vessels for each pig were weighed, and the weight of added sodium citrate was subtracted to record blood weight. Blood was subsampled in royal blue top trace element tubes with sodium heparin and stored at −20 °C for further dry matter (DM) and iron analysis. The stomach and the small and large intestines were cleaned of digesta by gently rinsing with water and dried by blotting with paper towels. The viscera and lung were stored together with the whole empty body. Organs (heart, liver, kidney, and spleen) and empty body (including the viscera) for each pig were weighed, subsampled, and stored at −20 °C for further DM and iron analysis. For subsampling, the middle portion of each organ (right kidney only), representing approximately one-fourth of the organ, was excised, and placed into a barrier bag. Collected whole empty body samples including the viscera (lung, stomach, small, and large intestine) were thawed for tissue homogenization via grinding in a meat grinder (M-22RW-20261-US, Torrey, Monterrey, Mexico), mixed again, subsampled, and stored at −20 °C until further DM and iron analysis.

### Blood parameter analysis

Collected plasma samples were analyzed for antioxidant parameters, including plasma catalase (CAT; Cayman Chemical Company, Ann Arbor, MI), superoxide dismutase (SOD; Cayman Chemical Company, Ann Arbor, MI), and malondialdehyde (MDA; Cayman Chemical Company, Ann Arbor, MI) using colorimetric assay kits and a spectrophotometer (Multiskan Skyhigh, Thermo Fisher Scientific, Waltham, MA) per manufacturer’s instructions.

### Iron analysis

Blood, colostrum, milk and tissue samples were analyzed for iron content at the Kansas Veterinary Diagnostic Laboratory (Manhattan, KS). Briefly, 1 g of the sample was digested in concentrated nitric acid (1.5 mL) and 30% hydrogen peroxide (0.5 mL-liver only) or concentrated nitric acid (1.5 mL) and 18 Ω water (1.5 mL-blood only) via microwave digestion (Multiwave Pro, Anton Paar, Ashland, VA). The microwave power was 1,500 W, and the following temperature program was used for digestion: from 0 to 15 min, the temperature was ramped to 165 °C then held at 165 °C for 15 min before cooling down to 70 °C in 16 min. Following digestion, the volume of the digested sample was adjusted to 10 mL with 18 Ω water. Additionally, a 1:1 dilution was prepared with 18 Ω water before analysis. A method blank containing all reagents, and no biological material was included in the run. Samples were diluted after digestion with 18 Ω water prior to analysis via inductively coupled plasma mass spectrometry (NexIon 350, Perkin Elmer, Shelton, CT). An 8-point calibration curve was built using standards in 2% aqueous nitric acid. The concentrations for iron ranged from 0.0064 ppm to 10 ppm. An internal standard solution containing Yttrium and Germanium was infused into each run (standards, samples, and blanks). Wet-basis iron content of each tissue, blood, and the whole empty body was multiplied by their respective weights to determine total iron mass in each component, and these values were summed to calculate whole body iron mass.

Tissue, blood, and whole empty body samples were analyzed for DM by weighing 5 g of each sample onto a weigh boat and dried in a drying oven (Fisherbrand™ Isotemp™ 650G Oven, Thermo Fisher Scientific, Waltham, MA) at 105 °C for 24 h. Final weight of each sample was then recorded to calculate moisture and DM. Data for iron content were expressed on a DM basis, except for colostrum and milk, which were expressed on an as-is basis.

### Statistical analysis

All data were analyzed with ANOVA for a randomized complete block design using PROC MIXED of SAS (version 9.2; SAS Inst. Inc., Cary, NC), except for colostrum and milk iron content that were analyzed with ANOVA for a completely randomized design using PROC MIXED of SAS. The experimental unit was an individual pig for growth performance in the suckling period, blood parameters in the entire suckling and nursery periods, and organ and whole-body iron content and mass; an individual sow for colostrum and milk iron content; and a pen for growth performance data in the nursery period. For the data from the suckling period, models included the iron injection, sire line, sire line × iron injection as fixed effects and the set within the sow litter and sow litter as random effects. For the data from the nursery period, models included the iron injection and sire line and their interaction as fixed effects and the replication as a random effect. For the colostrum and milk iron content, the models included the sire line as a fixed effect and the replication as a random effect. Outlier analysis within each treatment and day was performed using the Grubb’s test outlier calculator (GraphPad Software, San Diego, CA), and no outlier was identified. Least squares means were separated using the PDIFF option of SAS. Multiple comparisons were adjusted using the Tukey’s method. Statistical differences were considered significant at *P *< 0.05 and tendency at  *P *< 0.10.

## Results

### Growth performance

There were no sire line effects or interactions between sire line and iron injection frequency in growth performance during the suckling period (Table 2). Increasing iron injection frequency tended to linearly increase BW at d 15 of age (*P *= 0.07) and linearly increased BW at weaning (*P *< 0.05). Increasing iron injection frequency linearly increased ADG from d 8 to 15 of age (*P *< 0.05), from d 8 of age to weaning (*P *< 0.05), and from d 3 of age to weaning (*P *< 0.05) and tended to linearly increase ADG from d 3 to 15 of age (*P *= 0.06).

**Table 2:** Preweaning growth performance of pigs from two sire lines that received one- or two-iron injections during the suckling period.

	Sire^1^			Injection^2^				*P*-value				
Item	337	800	SEM	0	1	2	SEM	Sire	Injection	Interaction	L^3^	Q^4^
**Wean age**	19.57	18.47	0.19	18.89	18.88	19.00	0.24	–	–	–	–	–
**No. of pigs**	53	78	–	45	40	46	–	–	–	–	–	–
**Body weight, kg**												
** d 3 of age**	1.77	1.72	0.10	1.76	1.70	1.77	0.07	0.70	0.19	0.96	0.81	0.07
** d 8 of age**	2.51	2.62	0.19	2.56	2.51	2.63	0.14	0.68	0.33	0.94	0.41	0.22
** d 15 of age**	4.19	4.39	0.31	4.13	4.28	4.46	0.25	0.65	0.19	1.00	0.07	0.93
** Wean**	5.39	5.15	0.33	4.99	5.30	5.52	0.27	0.60	0.11	0.99	0.04	0.83
** Adj. wean^5^**	5.25	5.28	0.36	4.98	5.32	5.50	0.29	0.96	0.10	0.99	0.03	0.73
**Average daily gain, kg/d**												
** d 3–8 of age**	0.151	0.181	0.021	0.163	0.164	0.171	0.016	0.32	0.76	0.94	0.49	0.79
** d 8–15 of age**	0.239	0.252	0.022	0.223	0.252	0.262	0.018	0.68	0.05	1.00	0.02	0.51
** d 15 of age-wean**	0.249	0.224	0.016	0.204	0.252	0.255	0.017	0.27	0.03	0.73	0.01	0.23
** d 8 of age-wean**	0.246	0.245	0.020	0.220	0.255	0.261	0.017	0.97	0.03	0.93	0.01	0.31
** d 3–15 of age**	0.203	0.222	0.020	0.198	0.216	0.224	0.016	0.49	0.16	1.00	0.06	0.71
** d 3 of age-wean**	0.217	0.224	0.018	0.202	0.227	0.233	0.015	0.80	0.07	0.99	0.03	0.46
** Adj. d 3 of age-wean^5^**	0.219	0.222	0.018	0.202	0.226	0.233	0.015	0.89	0.08	0.99	0.03	0.47

1 337: PIC337 sire-line pigs; 800: PIC800 sire-line pigs.

2 Zero-iron injection: no iron injection in the suckling period; one-iron injection: 200 mg iron injection at d 3 of age and no additional iron injection in the suckling period; two-iron injection: 200 mg iron injections at d 3 of age and d 8 of age.

3 Linear effect with increasing iron injection frequency.

4 Quadratic effect with increasing iron injection frequency.

5 Adjusted weaning weight = body weight at d 3 of age + [(body weight at weaning − body weight at d 3 of age)/weaning age]  × 19 (Jang et al. 2017). Adjusted weaning weight was used for average daily gain calculation.

In the nursery period, PIC337 pigs had greater BW at d 0, 7, 14, and 21 (*P *< 0.05; Table 3) postweaning, and tended to have greater BW at d 28 (*P *= 0.06) postweaning than PIC800 pigs. PIC337 pigs had a significantly greater ADG from d 0 to 7 postweaning (*P *< 0.05) and ADFI from d 0 to 7 and from 0 to 14 postweaning (*P *< 0.05) and tended to have greater ADFI from d 7 to 14 postweaning (*P *= 0.08) and in overall nursery period (*P *= 0.10) and G:F ratio from d 0 to 7 postweaning (*P *= 0.09) compared to PIC800 pigs. Increasing iron injection frequency linearly increased BW at d 7, 14, 21 and 28 postweaning (*P *< 0.05), and ADG from d 7 to 14, from 14 to 21, from 0 to 14, and from 0 to 28 postweaning (*P *< 0.05). Increasing iron injection frequency linearly increased ADFI from d 7 to 14 and from 14 to 21 postweaning (*P *< 0.05) and tended to linearly increase from d 0 to 14 (*P *= 0.07), from 14 to 28 (*P *= 0.07), and from 0 to 28 postweaning (*P *= 0.06) with no interactions between sire line and iron treatment observed. Increasing iron injection frequency linearly increased G:F from d 7 to 14 and from 0 to 14 postweaning (*P *< 0.05). A significant interaction between sire line and iron injection frequency was observed in G:F from d 14 to 21 postweaning (*P *< 0.05), in which increasing iron injection frequency linearly decreased G:F linearly within PIC800 pigs, while linearly increasing within PIC337 pigs.

**Table 3:** Postweaning growth performance of pigs from two sire lines that received no, one-, or two-iron injections during the suckling period.^1^

	Sire^2^			Injection^3^				*P*-value				
Item	337	800	SEM	0	1	2	SEM	Sire	Injection	Interaction	L^4^	Q^5^
**Body weight, kg**												
** d 0 (wean)**	5.54	5.10	0.92	5.16	5.33	5.48	0.92	0.02	0.27	0.64	0.11	0.92
** d 7**	5.95	5.21	0.96	5.24	5.68	5.81	0.96	0.01	0.12	0.86	0.05	0.50
** d 14**	7.94	6.96	1.07	6.57	7.79	8.00	1.09	0.03	0.02	0.83	0.01	0.23
** d 21**	11.03	9.82	1.36	9.21	10.84	11.23	1.38	0.03	0.02	0.95	0.01	0.27
** d 28**	15.48	13.99	1.77	13.40	15.20	15.61	1.81	0.06	0.06	0.90	0.03	0.38
**Average daily gain, kg/d**												
** d 0–7**	0.059	0.015	0.014	0.013	0.050	0.048	0.02	0.05	0.27	0.62	0.18	0.36
** d 7–14**	0.284	0.251	0.023	0.189	0.300	0.312	0.03	0.32	0.02	0.53	0.01	0.17
** d 14–21**	0.443	0.408	0.043	0.378	0.437	0.462	0.04	0.19	0.05	0.81	0.02	0.53
** d 21–28**	0.636	0.596	0.061	0.598	0.623	0.627	0.06	0.34	0.81	0.85	0.56	0.80
** d 0–14**	0.171	0.133	0.017	0.101	0.175	0.180	0.02	0.13	0.03	0.93	0.02	0.18
** d 14–28**	0.539	0.502	0.051	0.488	0.530	0.544	0.05	0.26	0.35	0.94	0.17	0.69
** d 0–28**	0.355	0.318	0.032	0.295	0.353	0.362	0.03	0.15	0.09	0.97	0.05	0.37
**Average daily feed intake, kg/d**												
** d 0–7**	0.192	0.160	0.014	0.158	0.194	0.177	0.02	0.04	0.15	0.68	0.29	0.10
** d 7–14**	0.367	0.307	0.033	0.279	0.364	0.368	0.04	0.08	0.07	0.85	0.04	0.24
** d 14–21**	0.657	0.615	0.057	0.553	0.669	0.686	0.06	0.29	0.03	0.47	0.02	0.25
** d 21–28**	0.945	0.881	0.086	0.878	0.939	0.923	0.09	0.14	0.45	0.99	0.37	0.38
** d 0–14**	0.280	0.234	0.023	0.219	0.279	0.272	0.03	0.05	0.08	0.87	0.07	0.17
** d 14–28**	0.801	0.748	0.071	0.715	0.804	0.805	0.07	0.18	0.12	0.84	0.07	0.28
** d 0–28**	0.540	0.491	0.046	0.467	0.541	0.538	0.05	0.10	0.08	0.84	0.06	0.21
**Gain:feed**												
** d 0–7**	0.275	0.062	0.080	0.020	0.229	0.258	0.10	0.09	0.22	0.77	0.12	0.47
** d 7–14**	0.768	0.797	0.041	0.662	0.832	0.853	0.05	0.51	0.01	0.10	0.01	0.13
** d 14–21**	0.671	0.671	0.018	0.689	0.654	0.671	0.02	0.98	0.49	0.04^6^	0.54	0.31
** d 21–28**	0.672	0.673	0.015	0.683	0.661	0.675	0.02	0.97	0.70	0.54	0.74	0.44
** d 0–14**	0.600	0.540	0.042	0.432	0.623	0.656	0.05	0.34	0.02	0.67	0.01	0.24
** d 14–28**	0.672	0.671	0.011	0.684	0.658	0.673	0.01	0.95	0.44	0.15	0.57	0.26
** d 0–28**	0.655	0.645	0.012	0.632	0.649	0.669	0.02	0.61	0.28	0.35	0.12	0.95

1 *n *= 3 pens per treatment.

2 337: PIC337 sire-line pigs; 800: PIC800 sire-line pigs.

3 Zero-iron injection: no iron injection in the suckling period; one-iron injection: 200 mg iron injection at d 3 of age and no additional iron injection in the suckling period; two-iron injection: 200 mg iron injections at d 3 of age and d 8 of age.

4 Linear effect with increasing iron injection frequency.

5 Quadratic effect with increasing iron injection frequency.

6 Significant interactions between sire line and iron injection frequency, *P *< 0.05; means for zero, one- and two-iron injection treatments within PIC337 and PIC800 sire lines were 0.639, 0.673, 0.701, 0.738, 0.635, and 0.641, respectively.

### Hemoglobin levels

Hemoglobin levels did not differ by sire line at d 3 and 8 of age (*P *> 0.10; Table 4), while PIC800 pigs had greater hemoglobin levels (*P *< 0.05) at d 15 of age and weaning. Increasing iron injection frequency linearly and quadratically increased hemoglobin levels (*P *< 0.05) at d 8 and 15 of age, and at weaning with a peak value in the two-injection group, while no significant interaction was observed between sire line and iron injection frequency. Hemoglobin levels were significantly greater in PIC800 pigs at weaning (*P *< 0.05; Table 5) than PIC337 pigs although no difference was observed in the entire nursery period. Increasing iron injection frequency linearly and quadratically increased hemoglobin levels at d 0, 7, and 14 postweaning (*P *< 0.05) with a peak value in the two-injection group and at d 21 (*P *< 0.05) postweaning with a peak value in the one-injection group. Increasing iron injection frequency tended to quadratically increased hemoglobin levels at d 28 postweaning (*P *= 0.09) with a peak value in the one-injection group.

**Table 4:** Preweaning hemoglobin levels of piglets from two sire lines that received no, one-, or two-iron injections during the suckling period.

	Sire^1^			Injection^2^				*P*-value				
Item	337	800	SEM	0	1	2	SEM	Sire	Injection	Interaction	L^3^	Q^4^
**Hemoglobin, g/L**												
** No. of pigs**	53	78	–	45	40	46	–	–	–	–	–	–
** d 3 of age**	79.7	86.5	4.0	85.2	81.9	82.1	3.3	0.24	0.44	0.49	0.28	0.50
** d 8 of age**	87.2	91.3	2.2	78.1	93.7	95.9	2.1	0.19	<0.01	0.32	<0.01	0.01
** d 15 of age**	98.8	105.0	1.7	70.9	115.5	119.3	1.8	0.01	<0.01	0.20	<0.01	<0.01
** Wean**	96.3	103.9	2.5	66.0	114.0	120.3	2.3	0.04	<0.01	0.52	<0.01	<0.01

1 337: PIC337 sire-line pigs; 800: PIC800 sire-line pigs.

2 Zero-iron injection: no iron injection in the suckling period; one-iron injection: 200 mg iron injection at d 3 of age and no additional iron injection in the suckling period; two-iron injection: 200 mg iron injections at d 3 of age and d 8 of age.

3 Linear effect with increasing iron injection frequency.

4 Quadratic effect with increasing iron injection frequency.

**Table 5:** Postweaning hemoglobin levels of piglets from two sire lines that received no, one-, or two-iron injections during the suckling period.^1^

	Sire^2^			Injection^3^				*P*-value				
Item	337	800	SEM	0	1	2	SEM	Sire	Injection	Interaction	L^4^	Q^5^
**Hemoglobin, g/L**												
** d 0 (wean)**	93.5	104.8	3.6	64.8	113.9	118.6	4.2	0.03	<0.01	0.95	<0.01	<0.01
** d 7**	101.7	109.0	3.0	72.2	120.5	123.3	3.7	0.11	<0.01	0.64	<0.01	0.01
** d 14**	97.9	98.9	2.4	73.2	108.4	113.6	3.0	0.78	<0.01	0.28	<0.01	0.01
** d 21**	99.9	96.6	1.9	85.9	105.0	103.9	2.3	0.24	0.01	0.57	0.01	0.01
** d 28**	106.5	104.0	2.1	100.3	109.2	106.2	2.6	0.42	0.09	0.07	0.14	0.09

1 *n *= 11 pigs per treatment.

2 337: PIC337 sire-line pigs; 800: PIC800 sire-line pigs.

3 Zero-iron injection: no iron injection in the suckling period; one-iron injection: 200 mg iron injection at d 3 of age and no additional iron injection in the suckling period; two-iron injection: 200 mg iron injections at d 3 of age and d 8 of age.

4 Linear effect with increasing iron injection frequency.

5 Quadratic effect with increasing iron injection frequency.

### Iron content and retention

Colostrum and milk iron content were not different between sire lines (*P *> 0.10; Table 6). For iron content in pigs, PIC800 pigs had greater DM-basis whole empty body iron content than PIC337 pigs at birth (*P *< 0.05; Table 7). Whole iron mass in the liver of pigs at birth was significantly greater in PIC800 pigs than PIC337 pigs (*P *< 0.05), while no significant difference was observed in iron mass in the other organs, blood, and whole body between sire lines (*P *> 0.10). At weaning, there were no significant differences in DM-basis iron content of all organs, blood and whole empty body of pigs between sire lines (*P *> 0.10; Table 8). However, whole iron mass of the spleen tended to be greater in PIC337 pigs than PIC800 pigs (*P *= 0.09). Increasing iron injection frequency increased DM-basis iron content (*P *< 0.05) of blood, whole empty body, and all organs, linearly (and quadratically for the heart, liver, and spleen; *P *< 0.05). Increasing iron injection frequency also increased iron mass (*P *< 0.05) of blood, whole empty body, and all organs, linearly (and quadratically for the heart and liver; *P *< 0.05), resulting in a linear increase in whole body iron mass (*P *< 0.05). No interactions were observed between sire lines and iron injection frequency except for the heart (*P *< 0.05), where increasing iron injection frequency linearly increased iron mass within PIC800 pigs, while quadratically increasing within PIC337 pigs.

**Table 6:** Iron content in sow colostrum and milk (as-is basis).

	Sire^1^			*P*-value
Item	337	800	SEM	Sire
**Iron, ppm**				
** No. of samples**	7	8	–	–
** Colostrum^2^**	2.70	2.54	0.23	0.56
** Milk^3^**	2.47	2.33	0.25	0.62

1 337: PIC337 sire-line pigs; 800: PIC800 sire-line pigs.

2 Colostrum collected at farrowing (within 24 h postpartum).

3 Milk collected on d 15 of lactation.

**Table 7:** Iron content and whole iron mass in organs of piglets from two sire lines at birth before colostrum intake.^1^

	Sire^2^			*P*-value
Item	337	800	SEM	Sire
**Dry matter iron, ppm**				
** Heart**	204.45	194.43	18.01	0.71
** Liver**	450.03	344.97	175.73	0.61
** Kidney**	39.49	37.14	3.69	0.66
** Spleen**	556.89	612.76	70.53	0.60
** WEB^3^**	118.63	157.57	11.00	0.03
** Blood**	2,263.22	2,239.80	180.49	0.93
**Iron mass, mg**				
** Whole body**	46.96	55.15	3.99	0.21
** Heart**	0.37	0.38	0.03	0.79
** Liver**	1.95	5.54	1.34	0.04
** Kidney**	0.20	0.19	0.04	0.95
** Spleen**	0.16	0.20	0.02	0.33
** WEB^3^**	15.10	14.87	1.94	0.87
** Blood**	29.19	33.97	2.20	0.19

1 *n *= 6 pigs per sire line.

2 337: PIC337 sire-line pigs; 800: PIC800 sire-line pigs.

3 WEB: whole empty body including the small and large intestines and the lung.

**Table 8:** Iron content and whole iron mass in organs of weanling piglets from two sire lines that received no, one-, or two-iron injections during the suckling period.^1^

	Sire^2^			Injection^3^				*P*-value				
Item	337	800	SEM	0	1	2	SEM	Sire	Iron	Interaction	L^4^	Q^5^
**Dry matter iron, ppm**												
** Heart**	183.06	176.90	7.88	122.49	201.86	215.60	9.55	0.57	<0.01	0.13	<0.01	0.01
** Liver**	2,468.73	2,382.21	217.96	142.30	1,921.17	5,212.94	242.81	0.69	<0.01	0.41	<0.01	0.01
** Kidney**	222.97	223.58	14.15	132.92	214.70	322.21	17.33	0.98	<0.01	0.24	<0.01	0.55
** Spleen**	633.43	577.88	41.89	333.42	534.02	949.53	46.50	0.18	<0.01	0.35	<0.01	0.02
** WEB^6^**	117.01	108.02	13.44	57.04	115.93	164.67	14.98	0.50	<0.01	0.97	<0.01	0.72
** Blood**	2,034.21	1,913.59	82.47	1,566.19	2,053.73	2,301.79	101.00	0.31	<0.01	0.27	<0.01	0.34
**Iron mass, mg**												
** Whole body**	314.10	300.50	16.88	121.28	291.50	509.11	19.27	0.47	<0.01	0.78	<0.01	0.24
** Heart**	1.15	1.18	0.06	0.80	1.30	1.39	0.07	0.68	<0.01	0.04^7^	0.01	0.02
** Liver**	100.43	97.66	7.49	5.91	77.02	214.20	8.75	0.76	<0.01	0.52	0.01	0.01
** Kidney**	1.69	1.66	0.15	1.04	1.59	2.41	0.16	0.82	<0.01	0.32	0.01	0.38
** Spleen**	1.72	1.45	0.13	0.97	1.42	2.37	0.15	0.09	<0.01	0.60	0.01	0.12
** WEB^6^**	159.12	149.68	12.55	79.82	155.33	228.06	14.77	0.55	<0.01	0.90	0.01	0.93
** Blood**	49.97	48.89	3.37	32.74	54.86	60.70	4.13	0.82	<0.01	0.62	0.01	0.12

1 *n *= 6 pigs per treatment.

2 337: PIC337 sire-line pigs; 800: PIC800 sire-line pigs.

3 Zero-iron injection: no iron injection in the suckling period; one-iron injection: 200 mg iron injection at d 3 of age and no additional iron injection in the suckling period; two-iron injection: 200 mg iron injections at d 3 of age and d 8 of age.

4 Linear effect with increasing iron injection frequency.

5 Quadratic effect with increasing iron injection frequency.

6 WEB: whole empty body including small and large intestines and lung.

7 Significant interactions between sire line and iron injection frequency, *P *< 0.05; means for zero, one- and two-iron injection treatments within PIC337 and PIC800 sire lines were 0.75, 1.42, 1.27, 0.85, 1.17, and 1.51 mg, respectively.

There was no significant difference between two sire lines in plasma antioxidant and oxidative stress parameters (*P *> 0.10; Table 9). Increasing iron injection frequency tended to quadratically increase plasma MDA levels at weaning (*P = *0.06) and linearly and quadratically increased plasma MDA levels at d 14 postweaning (*P *< 0.05), with peak values observed in the one-injection group. Increasing iron injection frequency linearly and quadratically increased plasma CAT levels at d 0 postweaning (*P *< 0.05) with a peak value in the one-injection group and linearly increased at d 14 postweaning (*P < *0.05). Increasing iron injection frequency quadratically increased plasma SOD activity at d 14 postweaning (*P *< 0.05), with a peak value in the one-injection group.

**Table 9:** Post-weaning plasma antioxidant levels of piglets from two sire lines that received no, one-, or two-iron injections during the suckling period.^1^

	Sire^2^			Injection^3^				*P*-value				
Item	337	800	SEM	0	1	2	SEM	Sire	Iron	Interaction	L^4^	Q^5^
**Malondialdehyde, µM**												
** d 0**	11.72	12.06	0.38	10.55	12.64	12.49	0.47	0.54	0.01	0.89	0.01	0.06
** d 14**	15.55	14.83	0.40	13.79	16.21	15.57	0.49	0.21	0.01	0.78	0.02	0.02
** d 28**	6.81	7.46	0.42	7.32	7.05	7.03	0.49	0.20	0.86	0.44	0.63	0.82
**Catalase, nmol/min/mL**												
** d 0**	248.10	239.93	20.73	175.85	293.49	262.71	25.39	0.78	0.01	0.58	0.02	0.02
** d 14**	157.93	135.04	26.42	93.11	153.91	192.44	32.61	0.55	0.11	0.94	0.04	0.78
** d 28**	118.88	141.94	15.17	109.58	138.84	142.82	18.58	0.29	0.42	0.10	0.23	0.57
**Superoxide dismutase, U/mL**												
** d 0**	3.95	3.99	0.24	3.53	4.31	4.08	0.29	0.90	0.15	0.16	0.17	0.15
** d 14**	4.30	3.83	0.27	3.54	4.87	3.78	0.32	0.19	0.01	0.81	0.59	0.01
** d 28**	5.71	6.13	0.84	5.42	6.64	5.69	0.89	0.50	0.25	0.09	0.72	0.11

1 *n *= 6 pigs per treatment.

2 337: PIC337 sire-line pigs; 800: PIC800 sire-line pigs.

3 Zero-iron injection: no iron injection in the suckling period; one-iron injection: 200 mg iron injection at d 3 of age and no additional iron injection in the suckling period; two-iron injection: 200 mg iron injections at d 3 of age and d 8 of age.

4 Linear effect with increasing iron injection frequency.

5 Quadratic effect with increasing iron injection frequency.

## Discussion

Newborn pigs are susceptible to iron deficiency anemia as they are born with low iron reserves, and sow colostrum and milk contain a low amount of iron. With the recent advances in swine genetics for greater litter size and faster growth rate, suckling pigs may have increased iron demands during the suckling and nursery periods. This indicates that an additional iron injection may be needed before weaning to sufficiently meet their iron needs for growth and health (Chevalier et al. 2023; Johnson et al. 2025). In addition, there could be potential difference among various genetic lines and breeds in iron metabolism and retention and thereby hemoglobin levels, affecting pig growth and health (Egeli and Framstad 1998; Chevalier et al. 2024).

Although there was no significant difference observed in weaning weight and growth rate in the suckling period between sire lines, significant differences were observed in post-weaning growth performance between the PIC337 and PIC800 sire lines where PIC337 pigs had a greater BW, weight gain, and feed intake in the overall nursery period than PIC800 pigs with no interactions between sire line and iron injection frequency. In addition, PIC800 pigs had greater hemoglobin levels from the late suckling period to the early nursery period. These results indicate that hemoglobin levels in pigs can differ depending on genetics. This result agrees with Egeli and Framstad (1998) who reported greater hemoglobin levels were observed in Duroc pigs compared to Landrace pigs in the suckling period. In addition, Sun et al. (2025) reported that 53 quantitative trait loci are associated with the regulation of hematological traits in pigs, and traits, such as red blood cell count, are moderately to highly heritable in pigs, providing a strong genetic basis for this variation. Thus, these results indicate that iron metabolism and red blood cell production may be influenced by genetics, contributing to growth performance differences between sire lines. However, in this study, there was a difference in gestation length, resulting in weaning age a day shorter in PIC800 pigs compared with PIC337 pigs. This difference may result in numerical weaning weight differences, which may have an influence in post-weaning growth performance as increased weaning weight could increase BW and growth rate in the nursery period (Kwon et al. 2025). In addition, this increased weaning age may also have influenced these differences in post-weaning growth rate and feed intake (Main et al. 2004). Therefore, although there could be potential genetic influences in post-weaning pig growth, further studies are needed to clarify its effect with controlled weaning age and weight.

In addition, the absence of a sire line effect in pre-weaning growth suggests that genetic background may not be the major limiting factor for early life growth as iron injection frequency clearly led to growth differences in the suckling period. On the other hand, the sire line effect observed in post-weaning growth indicates that genetic variation between sire lines may play an important role in post-weaning growth, where metabolic differences in protein disposition and energy metabolism may be more pronounced (Elbert et al. 2020). However, it should be noted that PIC800 pigs had greater hemoglobin levels, but lower weaning weight compared with PIC337 pigs. This indicates that there could be potential influence in hemoglobin levels by body size and blood volume as hemoglobin levels are reported to be lower in pigs with heavy weaning weight compared with those with light weaning weight (Jolliff and Mahan 2011). Also, no sire line × injection interaction suggests that iron availability remains the dominant factor influencing hematological status in the early stages of life, although genetic factors could affect baseline hematological values rather than actual response to iron administration (Sun et al. 2025).

Increasing iron injection frequency increased pre-weaning weight gain linearly, resulting in a linear increase in weaning weight. This result indicates that iron deficiency due to no iron administration at birth resulted in poor growth performance even in the suckling period, which agreed with Chevalier et al. (2021). However, it has been reported that the response to an additional iron injection on growth performance may not appear in the suckling period although post-weaning growth performance could be improved by an additional iron injection (Albers et al. 2022; Johnson et al. 2025; Lima et al. 2025). This discrepancy in pre-weaning growth could result from differences in the additional iron injection timing, weaning age, baseline iron status, and genetic background of pigs. In the current study, the sire lines used were selected for faster growth rates and greater tissue accretion, which likely increased iron requirements earlier in the suckling period. This may explain the earlier growth response observed following an additional iron injection, with improvements in performance even before weaning. However, previous studies using traditional breeds (Landrace and Yorkshire crossbreed) reported 10–15% lower hemoglobin concentrations at weaning than those observed in the current study, despite receiving a single 200 mg iron injection (Chevalier et al. 2023; Johnson et al. 2025). Furthermore, Lima et al. (2025) reported that different iron injection strategies can influence pigs’ hematological and growth responses to supplemental iron during both suckling and nursery phases. However, because the mechanisms underlying the earlier growth response to an additional iron injection remain unclear, further research is needed to elucidate how pigs respond to administered iron under different injection strategies and how the responses affect their growth.

In this study, the linear increase in BW observed with increasing iron injection frequency in the late suckling period was maintained until the end of the nursery period and overall nursery weight gain increased linearly. These linear responses suggest that the amount of iron injected early in the suckling period provides a sufficient iron reserve able to support improved growth performance into the nursery period, which agrees with the previous studies (Chevalier et al. 2023; Johnson et al. 2025). In addition, pigs receiving no iron injection exhibited significantly reduced hemoglobin levels from weaning to the early nursery period, resulting in impaired growth performance, which agreed with previous studies (Williams et al. 2020; Chevalier et al. 2021). Interestingly, although growth performance and hemoglobin levels increased linearly with increasing iron injection frequency, the extent of improvement was substantially greater between no and one injection than between one and two injections. The significant rise in hemoglobin levels after one iron injection compared to no injection may indicate improved iron availability, leading to enhanced erythropoiesis (Ganz and Nemeth 2012). The diminished response between one and two iron injections likely reflects that pigs receiving one injection had already achieved relatively high hemoglobin levels, thereby limiting further increases in erythropoiesis. This may also be accompanied by elevated hepcidin expression in response to increased systemic iron, contributing to tighter regulation of iron availability (Lipinski et al. 2010). Therefore, these results suggest that body iron status and hemoglobin levels in pigs are limiting factors for growth in pigs under anemic conditions with no iron injection. However, once iron status is restored with an iron injection, iron availability may no longer be a growth-limiting factor, as the hemoglobin levels in pigs receiving one iron injection already exceeded the optimal levels (110 g/L) at weaning and only minimal increase (∼5%) were observed between one- and two-iron injection groups. As post-weaning growth was positively correlated with hemoglobin levels at weaning (Bhattarai and Nielsen 2015), the limited growth response to an additional iron injection likely indicates that pigs already had high hemoglobin levels after a single iron injection, thereby reducing the potential for further hemoglobin increases.

Increasing iron injection frequency resulted in linear increases in hemoglobin levels from late suckling period through d 14 postweaning. This result agrees with previous studies reporting that increasing iron injection frequency and dosage could increase hemoglobin levels linearly (Williams et al. 2020; Chevalier et al. 2021; Johnson et al. 2025; Lima et al. 2025). However, hemoglobin levels in pigs receiving no iron injection decreased from d 3 of age until weaning and increased after weaning as pigs consumed iron from the nursery diets although the hemoglobin levels were still under anemic range (< 90 g/L) until d 21 postweaning. These results indicate that rapid depletion of prenatal iron stores and low iron intake from sow milk alone in the suckling period are insufficient to support erythropoiesis and growth in the suckling and nursery periods. Similarly, previous studies reported that pigs receiving no iron injection during the suckling period showed hemoglobin levels below 90 g/L from d 3 of age until d 14 postweaning, resulting in reduced post-weaning growth performance, (Williams et al. 2020; Chevalier et al. 2021). It is important to note, however, that only by the end of the nursery period, pigs receiving no iron injection were able to reach hemoglobin levels within the suboptimal range (90–110 g/L). Although pigs obtained iron from nursery diets, allowing for partial recovery of hemoglobin levels, it took up to 4 wk to reach comparable hemoglobin levels to those in one- or two-iron injection group, which agrees with Johnson et al. (2025).

Regarding iron content and mass in various organs, blood, and whole body at birth and weaning along with iron content in colostrum and milk, no evident sire line effects were observed, despite sire line differences observed in hemoglobin levels in the suckling period. This result agrees with the previous study reporting that genetic background has minimal influence on tissue iron accumulation following parenteral iron administration (Egeli and Framstad 1998). However, Hermesch and Jones (2012) reported that muscle iron content exhibits moderate heritability and that genetic correlations between hematological traits and growth performance are generally weak, suggesting some genetic influence on iron-related traits. Overall, these findings indicate that while iron deposition in major body compartments may be relatively stable across genetic lines, certain tissue-specific iron status may still be under partial genetic control.

Iron injection frequency significantly increased iron content and whole mass in the liver, spleen, heart, and kidney at weaning, with the liver exhibiting the highest content and whole mass of iron among the collected organ samples. These results indicate that pigs in the suckling stage were able to efficiently incorporate the administered iron into storage in tissues, reflecting a direct relationship between iron supplementation and tissue iron contents. Johnson et al. (2025) demonstrated similar results, in which additional iron injection increased iron content in the liver. In addition, the highest content and mass of iron in the liver confirms the role of the liver as the primary site of iron storage in pigs, as reported by Chevalier et al. (2021, 2024), where the liver displayed the highest levels of iron among organs collected (Jensen et al. 2020). Interestingly, iron content and whole iron mass in circulating blood and the heart were similar between one- and two-injection groups, while they were linearly increased with increasing iron injection frequency in the other organs and whole body, highlighting differences in tissue-specific iron storage and metabolism. The relatively similar iron mass in blood between one and two injections is consistent with physiological regulation of circulating iron which is largely mediated by hepcidin. Hepcidin controls iron efflux by promoting degradation of ferroportin, thereby limiting further increases in serum iron once systemic iron levels rise (Collins et al. 2008; Ganz and Nemeth 2012). Cardiac tissue exhibits similar regulatory mechanisms, including local control by hepcidin and ferroportin, which prevents excess iron accumulation (Lakhal-Littleton et al. 2016) and may explain why heart iron content did not rise proportionally with increasing iron injection frequency from one to two in the current study. In contrast, organs with larger iron storage capacity, such as the liver and spleen, continue to sequester and store additional iron within ferritin, the major intracellular iron-storage protein, until it is needed by the body (Anderson and Frazer 2017). Overall, these findings demonstrate that tissue iron accumulation is dose-responsive, with organ iron content closely reflecting the quantity of iron administered during the suckling period.

In the current study, whole-body iron retention increased linearly with increasing iron injection frequency. Based on the results of the current study, pigs have approximately 51 mg of iron in their body at birth and accumulate an additional 70 mg of iron during the approximately 19-d suckling period when no iron injections were given. This accumulation is a result of the 2.4 to 2.6 ppm of iron in colostrum and milk, respectively. With one and two 200 mg iron injections, pigs retained nearly all of administered iron as the whole-body iron mass was 291.5 and 509.1 mg, representing increases of 170 and 388 mg, respectively compared with pigs that received no iron injection. Therefore, these results indicate that the majority of injected iron was retained once iron was administered and contributed directly to systemic iron status. Previously, Venn et al. (1947) reported an average whole-body iron mass of 46 mg in piglets at birth, which remains consistent with the findings of the current study. Thus, these findings in the current study provide updated information for iron reserve, accumulation, and requirements with modern genetic lines as it has been reported that pigs are born with 50 mg with 7–16 mg of iron required daily and sow milk contains 1 mg/L (Szudzik et al. 2018).

Iron is an essential component of many antioxidant enzymes, serving as a structural or catalytic cofactor. Catalase is a heme-containing enzyme that requires iron at its active site to catalyze the conversion of hydrogen peroxide into water and oxygen, which reduces the accumulation of reactive oxygen species (Chelikani et al. 2004). Similarly, SOD exists in iron- or other metal-containing forms (eg manganese and copper) and catalyzes the disproportionation of superoxide anions to reduce oxidative stress (Naranuntarat et al. 2009). In the current study, increasing iron injection frequency influenced systemic oxidative status, as reflected by changes in lipid peroxidation and antioxidant enzyme activity although there were no evident differences between two sire lines. Compared with pigs receiving no iron injection, pigs receiving one- or two-iron injections had increased plasma MDA levels at weaning and d 14 postweaning, resulting in quadratic responses with the peak values in pigs receiving one-iron injection. Meng et al. (2025) reported significantly greater serum MDA levels at d 21 of age in pigs receiving a single 200 mg of iron injection at d 3 of age compared with those receiving no iron injection. Circulating unbound iron can act as a pro-oxidant with the capacity to generate reactive hydroxyl radicals that result in lipid peroxidation (Lipiński et al. 2010; Gutteridge and Halliwell 2018). Thus, this result indicates that iron injection could increase oxidative stress in pigs with those receiving no iron injection.

However, with two-iron injections, pigs had slightly reduced plasma MDA levels compared with one-iron injection, indicating reduced oxidative stress at weaning and in the early nursery period with the additional iron injection. Similarly, Meng et al. (2025) reported significantly reduced serum MDA levels in pigs receiving additional oral iron supplementation (2.2 g iron per d) from d 2 to 13 of age along with a 200 mg of iron injection at d 3 of age compared with those receiving one iron injection. This result could be attributed to improved antioxidant enzyme levels as plasma CAT levels increased quadratically with increasing iron injection frequency with the peak values in the one-iron injection at weaning, while it linearly increased at d 14 postweaning. In addition, plasma SOD activity was quadratically increased with increasing iron injection frequency at weaning. Therefore, these results indicate that although iron injection could increase oxidative stress when compared with no iron injection, adequate iron availability is necessary to improve iron-dependent antioxidant system such as CAT and SOD (Chen et al. 2007; Zaka-Ur-Rab et al. 2016) and that iron deficiency can be associated with decreased activity of iron-dependent antioxidant enzymes.

## Conclusion

Increasing iron injection frequency during the suckling period enhanced, hemoglobin levels, pre- and post-weaning growth performance, plasma catalase activity, and whole-body iron retention in piglets, confirming that most of the injected iron was retained in the body. While sire line influenced growth performance and hemoglobin levels in piglets, it did not affect tissue and whole-body iron retention or antioxidant status in piglets.

## Abbreviations

ADFI: average daily feed intake
ADG: average daily gain
BW: body weight
C: control
CAT: catalase
DM: dry matter
G:F: gain-to-feed ratio
IM: intramuscular
MDA: malondialdehyde
SID: standardized ileal digestible
SOD: superoxide dismutase
STTD: standardized total tract digestible
WEB: whole empty body
